# Glabridin Ameliorates Alcohol-Caused Liver Damage by Reducing Oxidative Stress and Inflammation via p38 MAPK/Nrf2/NF-κB Pathway

**DOI:** 10.3390/nu15092157

**Published:** 2023-04-30

**Authors:** Mengyao Wang, Feng Zhang, Jie Zhou, Ke Gong, Shasha Chen, Xinran Zhu, Mengxue Zhang, Yajun Duan, Chenzhong Liao, Jihong Han, Zequn Yin

**Affiliations:** 1 College of Food and Biological Engineering, Hefei University of Technology, Hefei 230009, China; 2 Department of Cardiology, The First Affiliated Hospital of University of Science and Technology of China, Hefei 230001, China

**Keywords:** Glabridin, alcoholic liver injury, oxidative stress, inflammation, p38 MAPK, Nrf2, NF-κB

## Abstract

Licorice is a traditional and versatile herbal medicine and food. Glabridin (Gla) is a kind of isoflavone extracted from the licorice root, which has anti-obesity, anti-atherosclerotic, and antioxidative effects. Alcoholic liver disease (ALD) is a widespread liver disease induced by chronic alcohol consumption. However, studies demonstrating the effect of Gla on ALD are rare. The research explored the positive effect of Gla in C57BL/6J mice fed by the Lieber–DeCarli ethanol mice diet and HepG2 cells treated with ethanol. Gla alleviated ethanol-induced liver injury, including reducing liver vacuolation and lipid accumulation. The serum levels of inflammatory cytokines were decreased in the Gla-treated mice. The reactive oxygen species and apoptosis levels were attenuated and antioxidant enzyme activity levels were restored in ethanol-induced mice by Gla treatment. In vitro, Gla reduced ethanol-induced cytotoxicity, nuclear factor kappa B (NF-κB) nuclear translocation, and enhanced nuclear factor (erythroid-derived 2)-like 2 (Nrf2) nuclear translocation. Anisomycin (an agonist of p38 MAPK) eliminated the positive role of Gla on ethanol-caused oxidative stress and inflammation. On the whole, Gla can alleviate alcoholic liver damage via the p38 MAPK/Nrf2/NF-κB pathway and may be used as a novel health product or drug to potentially alleviate ALD.

## 1. Introduction

Drinking is a common dietary habit. Long-term drinking or bad drinking habits promote the onset and progression of the alcoholic liver [1]. Consistent with the Global Burden of Disease 2016, alcohol consumption is responsible for an estimated 2.8 million deaths, ranking seventh among the causes of death and disability worldwide [2]. Alcohol-induced liver damage includes liver steatosis, steatohepatitis, fibrosis to cirrhosis, and, ultimately, hepatic cancer [3]. Many factors may enhance the risk of alcoholic liver disease (ALD), including constant alcohol consumption, gender and race, family genetics, nutritional factors, smoking status, and so on, which can be mediated by hepatic oxidative stress, metabolic disorders, inflammation, and intestinal barrier dysfunction [4,5,6]. Ethanol is mainly metabolized in the liver. Primarily, ethanol is oxidized to acetaldehyde by ethanol dehydrogenase, then acetaldehyde is metabolized to acetate by aldehyde dehydrogenase [7]. Cytochrome P4502E1 (CYP2E1) also metabolizes ethanol into acetaldehyde at the endoplasmic reticulum and mitochondria [8]. Excessive ethanol intake also increases the expression and activity of CYP2E1, causing the excessive production of free radicals during the process. Excess free radicals cause oxidative damage which may result in alcoholic liver injury [9]. In addition, alcohol intake also promotes inflammation progression [10].

At present, the main way to treat ALD is still abstinence. In addition, drug treatment uses glucocorticoids, pentoxifylline, antioxidants, anti-TNF-α antibodies, etc., to improve hepatocyte damage, but there may be serious side effects [11]. It is also one of the exploration directions for researchers to find natural compounds with a low price and few side effects from food for ALD therapy.

Licorice is a species of the legume plant, mainly divided into *Glycyrrhiza uralensis Fisch*, *Glycyrrhiza inflata Bat*, and *Glycyrrhiza glabra* L. Licorice root has a long history of consumption and is still used as a dietary supplement to improve health in many regions [12]. Many studies have demonstrated the hepatoprotective effects of licorice; for example, glycyrrhizin alleviated endotoxin-induced acute liver injury [13], the ethanol extract of licorice attenuated high-fat-diet-induced liver lipid accumulation [14], isoliquiritigenin activated the peroxisome proliferative-activated receptor gamma coactivator 1-alpha to alleviate alcoholic liver injury [15], etc. Glabridin (Gla), a kind of isoflavone, is derived from *G. glabra* L. roots, and has antioxidant, anti-inflammatory, and anti-atherogenic effects [16]. Joo-Won Lee et al. found that Gla may have ameliorated high-fat-diet-induced obesity by the AMP-activated protein kinase [17]. In addition, Gla also improved liver injury caused by methotrexate, mainly through decreasing oxidative stress and inflammation [18]. Although the hepatoprotective effect of Gla has been demonstrated, the role of Gla is still unknown in alcoholic liver injury. Hence, we explored the protective role of Gla on ALD model mice, and elucidated its potential mechanism, which may provide another effective functional dietary supplement for patients with alcoholic liver injury.

## 2. Materials and Methods

### 2.1. Reagents

Glabridin (Gla, standard purity was 97%, Cat# 59870-68-7) was obtained from Bidepharm (Shanghai, China). Methylcyclopentadienyl Manganese Tricarbonyl (MTT) was obtained from Sigma-Aldrich (Cat# CT01, St Louis, MO, USA). NLR family, pyrin domain containing 3 (NLRP3, Cat# A5652), PYD and CARD domain containing (ASC, Cat# A1170), caspase-1 (Cat# A0964), tubulin, alpha 1b (a-tublin, Cat# HRP-66031), Lamin A/C (Cat# A0249), nuclear factor kappa B (NF-κB, Cat# A16271), superoxide dismutase 1 (SOD1, Cat# A0274), glyceraldehyde-3-phosphate dehydrogenase (GAPDH, Cat# AC033), p38 MAPK (Cat# A14401), phosphorylated-p38 MAPK (p-p38 MAPK, Cat# AP0526), extracellular signal-regulated kinase (p-ERK/ERK, Cat# A16686/AP0472), and c-jun N-terminal kinase (p-JNK/JNK, Cat# A4867/AP0631) antibodies were obtained from ABclonal (Boston, MA, USA). Interleukin 1β (IL-1β, Cat# 16806-1-AP), CYP2E1 (Cat# 19937-1-AP), and tumor necrosis factor alpha (TNF-α, Cat# 60291-1-lg) polyclonal antibodies were obtained from Proteintech (Chicago, IL, USA). Anti-phosphorylated-NF-κB (p-NF-κB, Cat# AF2006), interleukin 6 (IL-6, Cat# DF6087), and nuclear factor (erythroid-derived 2)-like 2 (Nrf2, Cat# AF0639) polyclonal antibodies were purchased from Affinity Biosciences (Cincinnati, OH, USA). Rabbit IgG (Cat# 2729) was obtained from Cell Signaling Technology (Boston, MA, USA). TNF-α, IL-1β, and IL-6 ELISA kits (Cat# RX203371M, RX203063M, and RX-G203049M) were obtained from Ruixinbio (Quanzhou, China).

### 2.2. Animals

Animal research was processed in accordance with the protocols approved by the Animal Ethics Committee of the Hefei University of Technology (approval number: HFUT20220315001).

C57BL/6J female mice (~10 weeks old, ~22 g) were obtained from GemPharmatech (Nanjing, China) and placed in plastic cages with corncob bedding material at least 5 days before experiments, with up to 5 mice per cage. All the animals were maintained in an environment of 23 ± 1 °C, relative humidity 60–70%, and light/dark cycle of 12 h, free to obtain water and food.

A short-term chronic ethanol-fed was used for constructing an ALD model in female mice according to previously published literature [19]. Initially, all the mice adapted to a liquid diet by feeding them a Lieber–DeCarli regular control diet for 5 days. Then, mice were continuously fed a Lieber–DeCarli ethanol mice diet containing 5% (vol/vol) ethanol for 10 days. On the eleventh day, the mice were given gavage of ethanol (5 g/kg). The control groups were given a Lieber–DeCarli regular control diet with gavage of isocaloric maltose dextrin solution. Lieber–DeCarli regular control diet (Cat# 710027) and Lieber–DeCarli ethanol mice diet (Cat# 710260) were purchased from Dyets (Wuxi, China). The composition and contents of the two diets were shown in Table S1.

The effective dose of Gla administered orally to mice was determined based on previously described literature [18]. We split the female mice into four groups at random: (1) pair-fed diet with gavage of 0.5% carboxymethylcellulose daily (Con); (2) pair-fed diet with Gla (40 mg/kg, i.g) daily (Gla); (3) EtOH-fed diet with gavage of 0.5% carboxymethylcellulose daily (EtOH); and (4) EtOH-fed diet with Gla (40 mg/kg, i.g) daily (EtOH + Gla). All the mice were euthanized 9 h after gavage of ethanol or maltose dextrin and liver and serum was saved at −80 °C.

### 2.3. Histopathologic Assay

Mouse liver tissues were collected, fixed with 4% paraformaldehyde for more than 24 h, embedded in the paraffin, and sliced into 5 mm sections. Paraffin sections were used for H&E staining. Fixed liver tissues were dehydrated with 30% sucrose and then sliced into 5 μm frozen sections for dihydroethidium (DHE) staining, immunofluorescence staining, and Oil Red O staining according to a previous study [20]. At the same time, a negative control is necessary in each immunofluorescence experiment. All the images were observed with a Leica fluorescence microscope (Leica, Wetzlar, Germany). Later, the mean fluorescence intensity (MFI) was analyzed by Image J 1.8.0 software.

### 2.4. Determination of Liver TG, FFA, GSH, SOD, and CAT Levels

Approximately 30 mg liver tissue was ground into homogenate to detect triglyceride (TG), free fatty acid (FFA), glutathione (GSH), SOD, and catalase (CAT) levels by TG content detection kit (Cat# BC0625), FFA content detection kit (Cat# BC0595), reduced GSH assay kit (Cat# A006-2-1), SOD assay kit (Cat# A001-3-2), and CAT assay kit (Cat# A007-1-1), which were from Solarbio (Beijing, China) and Nanjing Jiancheng Bioengineering Insititute (Nanjing, China).

### 2.5. Assessment of Serum Parameters

The blood samples of mice were left standing at room temperature for least 2 h, and then centrifuged for 20 min at 2000× *g* subsequently, and the serum in the supernatant was obtained and stored at −80 °C. The levels of alanine aminotransferase (ALT) and aspartate aminotransferase (AST) in the mice serum were tested by an automatic biochemical analyzer (Cat# 3100, Hitachi high-tech Corporation, Tokyo, Japan). Serum levels of inflammatory cytokines (TNF-α, IL-1β, and IL-6) in mice were assessed by ELISA.

### 2.6. Reactive Oxygen Species (ROS) Level Determination

In HepG2 cells, the ROS levels were detected by 2′,7′-Dichlorodihydrofluorescein diacetate (DCFH-DA) method mentioned in the past study [21]. Simply, HepG2 cells in a 6-well plate were incubated with DCFH-DA for 20 min and later washed 3 times with PBS. The ROS levels of the mouse liver were determined by DHE staining. Briefly, the liver’s frozen sections were thawed for 30 min at room temperature and immersed in PBS for 5 min, and then incubated in 5 mM DHE solution at room temperature for 60 min. The fluorescence identity was observed and photographed with a fluorescent microscope under dark conditions.

### 2.7. TUNEL Staining

Apoptosis in the liver was detected using liver’s frozen sections by the TUNEL apoptosis kit (Vazyme Biotechnology, Nanjing, China). Images were obtained from a fluorescence microscope under dark conditions.

### 2.8. Western Blot and Quantitative Real-Time PCR (qPCR)

Cell cultures or 30 mg mouse liver tissue was lysed using protein lysis buffer supplemented with protease inhibitors. BCA protein assay reagent kit (Thermo Fisher Scientific, Waltham, MA, USA) was used for detecting protein concentration. All densitometric data for the target protein were normalized with GAPDH and a-tublin, which were used as the loading controls.

Trizol reagent (Beijing Zomen Biotechnology, Beijing, China) was used for RNA extraction of liver tissues and cells. A reverse transcription kit (New England Biolabs, Ipswich, MA, USA) was used for cDNA synthesis. Then, qPCR was performed on LightCycler96 (Roche, Mannheim, Baden-Württemberg, Germany) with the primers exhibited in Table 1. β-actin and GAPDH were selected as the housekeeping genes.

### 2.9. Cell Culture

HepG2 cells were obtained from ATCC (Rockville, MD, USA) and cultured in MEM medium (Biological Industries, Kibbutz Beit-Haemek, Israel), involved with 10% fetal bovine serum and 1% penicillin (100 U/mL)–streptomycin (100 U/mL), and maintained in a humidified incubator with 95% air and 5% CO_2_ at 37 °C. HepG2 cells were treated with EtOH (100 mM) and Gla (8 mM) for 24 h. HepG2 cells were pretreated with Anisomycin (an agonist of p38 MAPK [22], 50 ng/mL) for 30 min and then treated with EtOH (100 mM) and Gla (8 mM) for 24 h.

### 2.10. Cell Viability Detection

The viability of HepG2 cells was determined by MTT assay. Simply, after treating, HepG2 cells were incubated with MTT (0.5 mg/mL) for 4 h and later cultured in dimethyl sulfoxide for another 15 min. The plate was placed in a shaker to oscillate and mix at low speed. The absorbance wavelength at 490 nm was detected by the microplate reader (BioTek SYNERGYH1, Winooski, VT, USA).

### 2.11. Statistical Analysis

The data were exhibited as the mean ± standard deviation. Two-way analysis of variance was adopted for statistical comparisons. A significant difference was considered if *p* < 0.05 (*n* ≥ 3). All the experiments were repeated three or more times. Statistical analysis was operated on GraphPad Prism 8.

## 3. Results

### 3.1. Gla Ameliorated Liver Injury in Ethanol-Induced Mice

**Figure 1:**
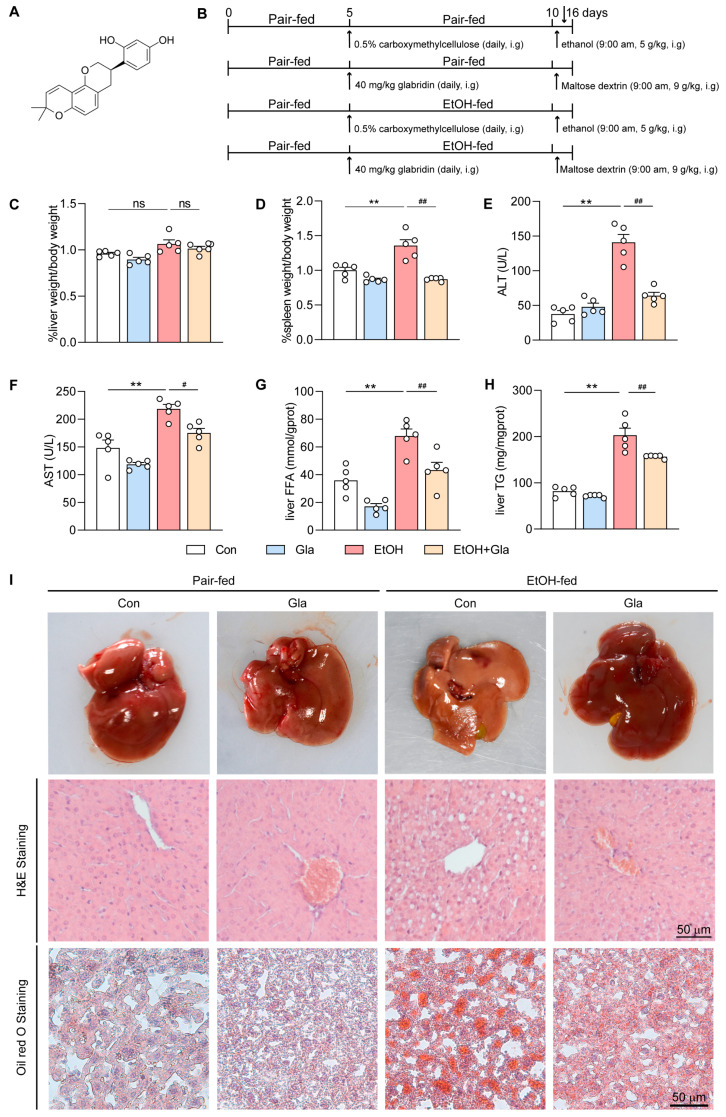
Gla ameliorated ethanol-induced liver injury in vivo. (**A**) Chemical structure of Gla. (**B**) The design of the experiment in vivo. (**C**,**D**) The ratio of liver-to-body weight and spleen-to-body weight in the mice (*n* = 5). (**E**,**F**) Serum ALT and AST levels (*n* = 5). (**G**,**H**) FFA and TG levels (*n* = 5). (**I**) Liver photographs (top); H&E staining (middle); Oil red O staining (bottom). ns, not significant; ** *p* < 0.01 vs. “Con” group; ^#^ *p* < 0.05, ^##^ *p* < 0.01 vs. “EtOH” group.

The chemical structure of Gla is shown in Figure 1A. To explore the effect of Gla on ALD, the female mice were split into four groups. Subsequently, the four groups were treated as shown in Figure 1B, euthanized on the last day, and the tissues and serum were collected. After Gla treatment, Gla could lower the elevated liver-to-body weight slightly (Figure 1C) and the spleen-to-body weight ratio (Figure 1D) significantly, induced by the ethanol diet, indicating that the function of Gla was possibly associated with the inflammation induced by ethanol. ALT and AST are vital markers reflecting liver damage. According to Figure 1E,F, serum levels of ALT and AST were significantly elevated in the EtOH group, but reduced by Gla treatment. We also detected via ELISA that Gla could reduce the levels of hepatic TG and FFA (Figure 1G,H). In Figure 1I, the liver showed a normal reddish-brown color and the hepatocytes were arranged neatly and closely in the control group. Inversely, the liver in the EtOH group was white, and the hepatocyte arrangement was looser than the control group. We also observed enlarged vacuoles and more severe lipid accumulation in the EtOH group. Liver morphology was significantly improved and vacuoles and lipid droplets were reduced after Gla treatment. Taken together, these results indicated that Gla can obviously improve liver injury caused by an ethanol diet.

### 3.2. Effect of Gla on Inflammation of Ethanol-Induced Mice

**Figure 2:**
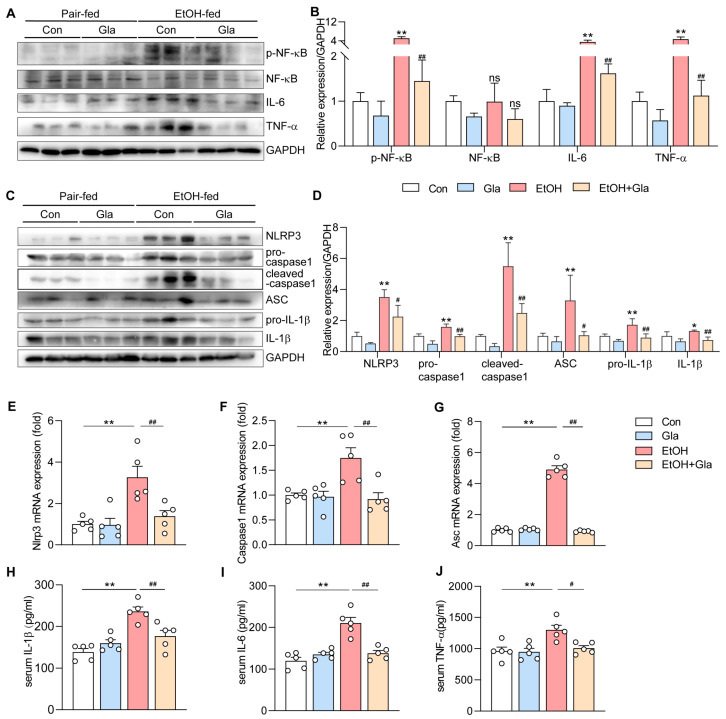
Gla ameliorated liver inflammation induced by ethanol via activating the NF-κB pathway in vivo. (**A**–**D**) Protein levels of p-NF-κB, NF-κB, IL-6, TNF-α, NLRP3, pro-caspase1, cleaved-caspase1, ASC, pro-IL-1β, and IL-1β were determined using Western blot method (*n* = 6). (**E**–**G**) mRNA levels of Nlrp3, Caspase1, and Asc were determined by qPCR (*n* = 5). (**H**–**J**) Serum levels of IL-1β, IL-6, and TNF-α (*n* = 5). ns, not significant; * *p* < 0.05; ** *p* < 0.01 vs. “Con” group; ^#^ *p* < 0.05, ^##^ *p* < 0.01 vs. “EtOH” group.

Alcohol intake contributes to the progression of liver inflammation [23].Thus, we investigated the influence of Gla on the inflammation involved with the NF-κB pathway in ALD. The protein levels of p-NF-κB, IL-6, and TNF-α in the mouse liver of the EtOH group were increased compared to the control group, which were significantly reduced by Gla treatment (Figure 2A,B). The levels of NLRP3, pro-caspase1, cleaved-caspase1, ASC, pro-IL-1β, and IL-1β in the mouse liver of the EtOH group were elevated, but the levels of the above proteins were decreased as determined by a Western blot after Gla treatment (Figure 2C,D). Moreover, NLRP3, Caspase-1, and ASC were also decreased by Gla in the transcriptional level (Figure 2E–G). In addition, the results revealed that Gla significantly lowered the serum levels of IL-1β, IL-6, and TNF-α in the EtOH-fed mice (Figure 2H–J). On the whole, the above results suggested that Gla evidently reduced the ethanol-induced inflammatory responses in mice.

### 3.3. Effect of Gla on Oxidative Stress and Apoptosis of Ethanol-Induced Mice

**Figure 3:**
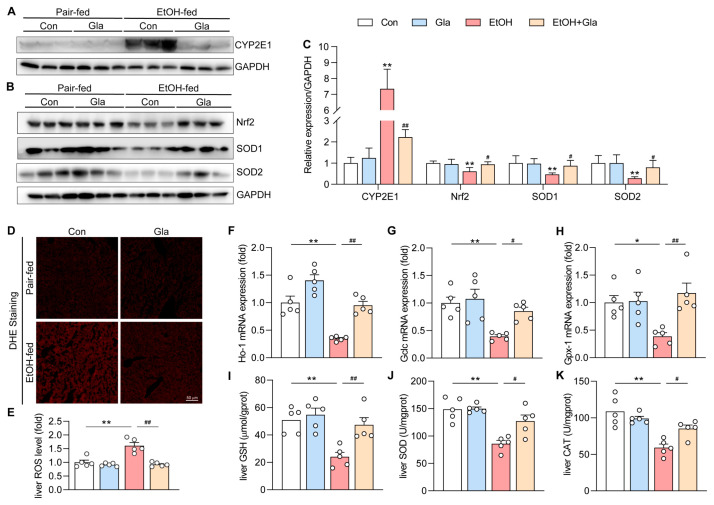
Gla ameliorated liver oxidative stress caused by ethanol via the Nrf2 pathway in vivo. (**A**–**C**) Protein levels of CYP2E1, Nrf2, SOD1, and SOD2 were detected by Western blot (*n* = 6). (**D**,**E**) DHE staining and its statistics (*n* = 5). (**F**–**H**) mRNA levels of Ho-1, Gclc, and Gpx-1 were detected by qPCR (*n* = 5). (**I**–**K**) Content levels of liver GSH and enzyme activity levels of SOD and CAT (*n* = 5). * *p* < 0.05, ** *p* < 0.01 vs. “Con” group; ^#^ *p* < 0.05, ^##^ *p* < 0.01 vs. “EtOH” group.

**Figure 4:**
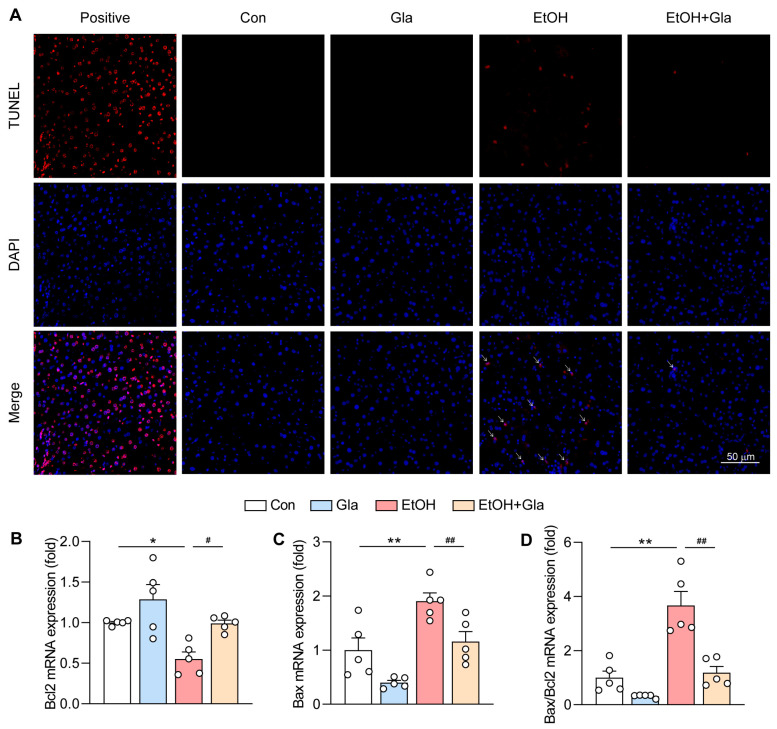
Gla ameliorated liver apoptosis induced by ethanol in vivo. (**A**) TUNEL staining for apoptosis detection. (**B**–**D**) mRNA levels of Bcl2, Bax, and Bax/Bcl2 were detetected by qPCR (*n* = 5). * *p* < 0.05, ** *p* < 0.01 vs. “Con” group; ^#^ *p* < 0.05, ^##^ *p* < 0.01 vs. “EtOH” group.

Excess ethanol will be metabolized by CYP2E1, thus producing a large number of ROS, resulting in oxidative damage and apoptosis. To study the role of Gla on the ethanol-caused liver oxidative stress, we detected CYP2E1 and the Nrf2 pathway in the molecular level. We observed that Gla treatment reduced the level of CYP2E1, while increasing the levels of Nrf2, SOD1, and SOD2 (Figure 3A–C). In the EtOH group mice, the expression of antioxidant genes including Ho-1, Gclc, and Gpx-1 was also increased after Gla treatment as determined by qPCR (Figure 3F–H). In addition, Gla could decrease ethanol-induced ROS levels determined by DHE staining in the liver (Figure 3D,E). Additionally, our findings indicated that ethanol feeding diminished the content of GSH and activities of SOD and CAT in the mouse liver, while Gla treatment restored these indicators (Figure 3I–K). Subsequently, we detected apoptosis by TUNEL staining and found that Gla effectively inhibited the increase of apoptotic cells induced by ethanol feeding in the mouse liver (Figure 4A). Accordingly, Gla reduced the Bax and Bax/Bcl2 mRNA levels and increased the Bcl2 mRNA level in vivo (Figure 4B–D). Taken together, we indicated that Gla can alleviate oxidative stress and apoptosis of ethanol-feeding mice.

### 3.4. Effect of Gla on HepG2 Cell Viability

**Figure 5:**
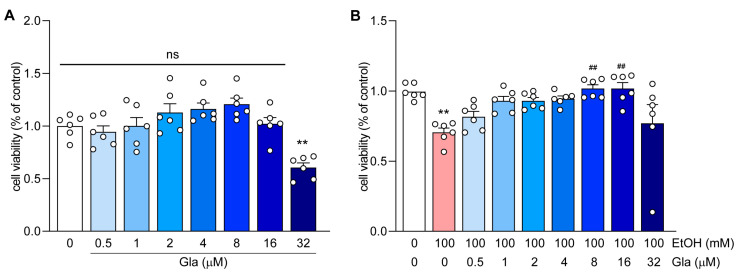
Gla restored HepG2 cell viability induced by ethanol in vitro. (**A**) Cell viability of HepG2 cells incubated with Gla at the concentrations (0, 0.5, 1, 2, 4, 8, 16, and 32 mM) for 24 h (*n* = 6). (**B**) Cell viability of HepG2 cells incubated with Gla at the concentrations (0, 0.5, 1, 2, 4, 8, 16, and 32 mM) and EtOH (100 mM) together for 24 h (*n* = 6). ns, not significant; ** *p* < 0.01 vs. “0” group; ^##^ *p* < 0.01 vs. “100 mM EtOH” group.

Firstly, we detected the toxicity of Gla on HepG2 cells by the MTT method. As shown in Figure 5A, the concentration of Gla less than or equal to 8 mM shows almost no toxicity toward HepG2 cells. Later, we constructed an alcoholic liver model in HepG2 cells in vitro according to a previous study [24]. We noticed that a 100 mM ethanol treatment alone evidently decreased cell viability, but the combined treatment with 8 mM and 16 mM Gla both significantly rescued the phenomenon (Figure 5B). Therefore, 8 mM of Gla was chosen as the experimental dose in later tests. Taken together, Gla significantly prevented ethanol-induced cell damage.

### 3.5. Gla Attenuated the NF-κB-Mediated Inflammation in Ethanol-Induced HepG2 Cells

**Figure 6:**
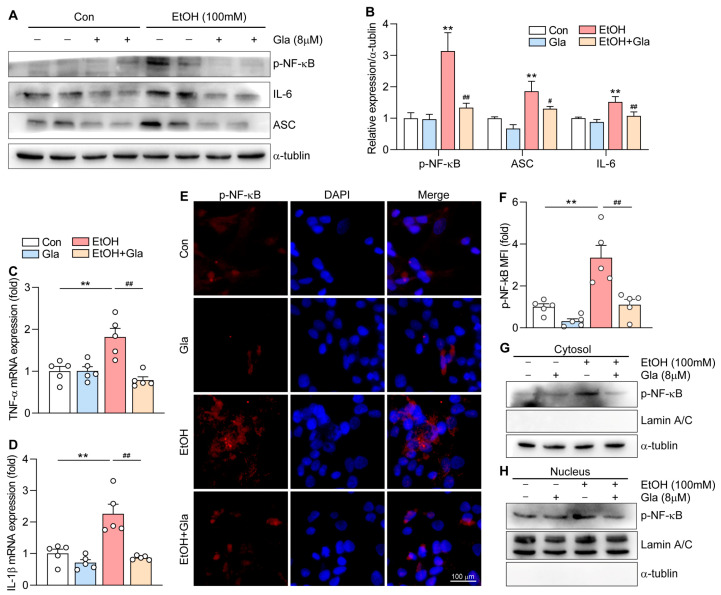
Gla ameliorated inflammation induced by ethanol by inhibiting the NF-κB pathway in vitro. HepG2 cells were incubated with EtOH (100 mM) and Gla (8 mM) for 24 h. (**A**,**B**) Protein levels of p-NF-κB, IL-6, and ASC were detected by Western blot (*n* = 3). (**C**,**D**) mRNA levels of TNF-a and IL-1β were detected by qPCR (*n* = 5). (**E**) Protein expression of p-NF-κB was determined using immunofluorescent staining method. (**F**) MFI statistical histogram of p-NF-κB (*n* = 5). (**G**–**H**) Protein levels of p-NF-κB in the cytosol and nucleus were determined using Western blot method (*n* = 3). **p < 0.01 vs. “Con” group; ^#^ *p* < 0.05, ^##^ *p* < 0.01 vs. “EtOH” group. MFI, mean fluorescence intensity.

Previously, we proved that Gla can relieve the inflammation caused by ethanol in vivo; hence, we explored the influence of Gla on the inflammation in HepG2 cells. The findings suggested that Gla can decrease the expression of p-NF-κB, IL-6, and ASC detected using the Western blot method, and TNF-α and IL-1β as determined by qPCR in HepG2 cells treated with ethanol (Figure 6A–D). Subsequently, we detected the nuclear translocation of p-NF-kB by immunofluorescence. The expression of p-NF-κB was increased especially in the nucleus. The MFI of p-NF-κB was reduced (Figure 6E,F) after Gla treatment. Similar results were obtained from the nuclear protein lysates and cytoplasmic lysates of HepG2 cells (Figure 6G,H). Taken together, Gla may alleviate inflammation induced by ethanol in HepG2 cells by inhibiting the NF-κB pathway.

### 3.6. Gla Promoted the Nrf2-Mediated Antioxidant Responses in Ethanol-Induced HepG2 Cells

**Figure 7:**
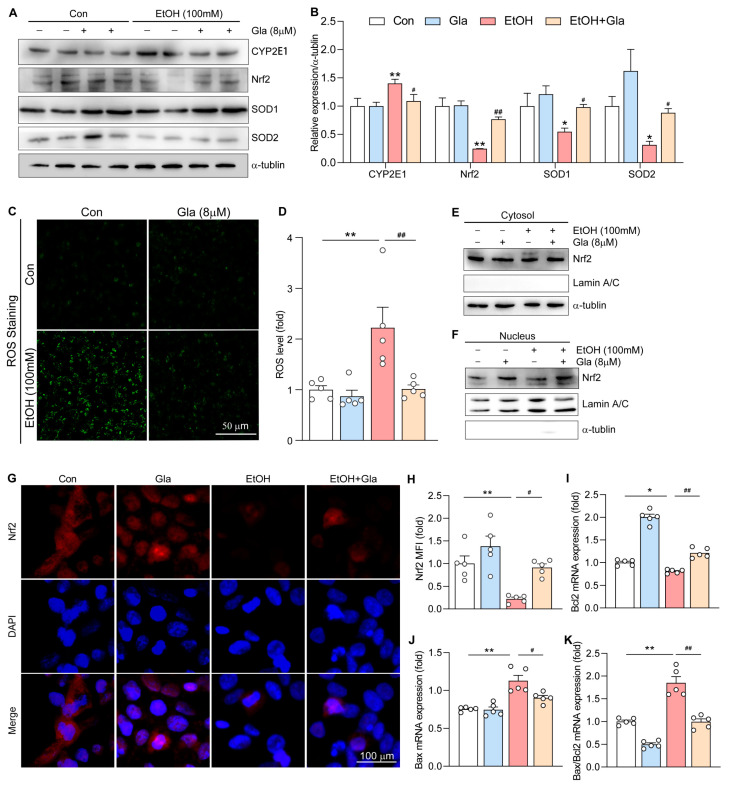
Gla ameliorated oxidative stress induced by ethanol via the Nrf2 pathway in vitro. HepG2 cells were incubated with EtOH (100 mM) and Gla (8 mM) for 24 h. (**A**,**B**) Protein levels of CYP2E1, Nrf2, SOD1, and SOD2 were detected using Western blot method (*n* = 3). (**C**,**D**) DCFH-DA staining and its statistics (*n* = 5). (**E**,**F**) Protein levels of Nrf2 in the cytosol and nucleus were detected using Western blot method (*n* = 3). (**G**) Protein expression of Nrf2 was determined by immunofluorescent staining. (**H**) MFI statistical histogram of Nrf2 (*n* = 5). (**I**–**K**) mRNA levels of Bcl2, Bax, and Bax/Bcl2 were detected by qPCR (*n* = 5). * *p* < 0.05, ** *p* < 0.01 vs. “Con” group; ^#^ *p* < 0.05, ^##^ *p* < 0.01 vs. “EtOH” group.

Consistent with animal experiment results, Gla evidently decreased the expression of CYP2E1 but promoted the expression of Nrf2, SOD1, and SOD2 in HepG2 cells treated with ethanol (Figure 7A,B). Moreover, ROS staining results indicated that Gla played a significant inhibitory role on ethanol-caused oxidative stress in HepG cells (Figure 7C,D). Similarly, we extracted the nuclear and cytoplasmic proteins of HepG2 cells for Western blot detection, and indicated that Gla could evidently increase the level of Nrf2 in the nuclear proteins (Figure 7E,F). Immunofluorescence results also showed that Gla could restore Nrf2 levels, especially in the nucleus (Figure 7G,H). Gla was previously shown to reduce ethanol-induced apoptosis in mouse hepatocytes. Consistently, the mRNA levels of Bcl2 and Bax were decreased by Gla in HepG2 cells after ethanol treatment (Figure 7I–K). Taken together, Gla may play an antioxidant role by activating the Nrf2 pathway.

### 3.7. Gla May Ameliorate Ethanol-Induced Oxidative Stress and Inflammation via the p38 MAPK/Nrf2/NF-κB Pathway

**Figure 8:**
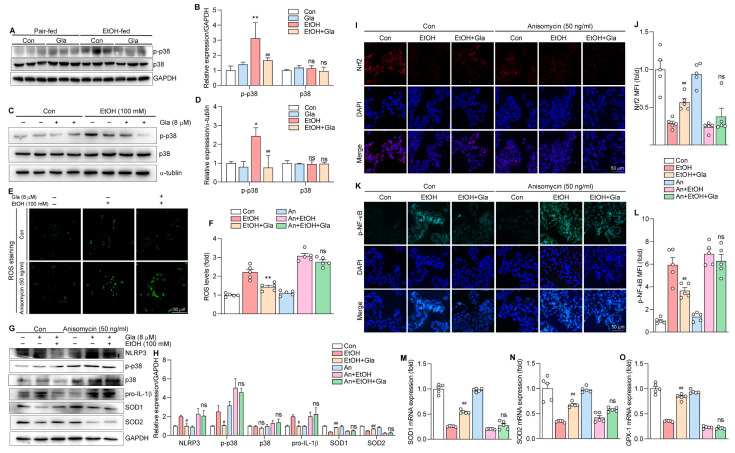
Gla ameliorated ethanol-induced oxidative stress and inflammation by p38/Nrf2/NF-κB signaling pathway in vitro. (**A**,**B**) Protein levels of p-p38/p38 in the mouse liver were determined using Western blot method (*n* = 6). (**C**,**D**) Protein levels of p-p38/p38 were detected by Western blot in HepG2 cells treated with EtOH (100 mM) and Gla (8 mM) for 24 h (*n* = 3). HepG2 cells in (**E**–**O**) were pretreated with Anisomycin (a p38 MAPK agonist, 50 ng/mL) for 30 min, and then treated with EtOH (100 mM) and Gla (8 mM) for 24 h. (**E**,**F**) DCFH-DA staining for ROS level detection (*n* = 5). (**G**,**H**) Protein levels of NLRP3, p-p38, p38, pro-IL-1β, SOD1, and SOD2 were determined by Western blot (*n* = 3). (**I**,**K**) Protein expression of Nrf2 and p-NF-κB was determined by immunofluorescent staining. (**J**,**L**) MFI statistical histograms of Nrf2 and p-NF-κB (*n* = 5). (**M**,**O**) mRNA levels of SOD1, SOD2, and GPX-1 were determined by qPCR (*n* = 5). * *p* < 0.05, ** *p* < 0.01 vs. “Con” group; ^#^ *p* < 0.05, ^##^ *p* < 0.01 vs. “EtOH” group; ns, no significant; ns vs. “An + EtOH” group.

P38 MAPK as a common protein kinase regulates many biological processes, such as inflammation related to the NF-κB pathway and oxidative stress related to the Nrf2 pathway [25,26]. These results showed that the level of p-p38 was evidently induced by ethanol but was decreased by Gla treatment in vitro and in vivo (Figure 8A–D). Thus, we supposed that p38 MAPK plays key roles in the anti-inflammatory and antioxidative effects of Gla. To investigate whether Gla regulates the oxidative stress mediated by Nrf2 and the inflammation mediated by NF-κB through p38 MAPK, we treated HepG2 cells with the Anisomycin (50 ng/mL, a p38 MAPK agonist) together with ethanol and Gla. The results suggested that Gla could no longer significantly reduce ethanol-induced high ROS levels when p38 MAPK was strongly activated by Anisomycin (Figure 8E,F). As shown in Figure 8G, H, M, N, and O, in the absence of Anisomycin, Gla obviously decreased the levels of NLRP3 and pro-IL-1β, and enhanced the levels of SOD1, SOD2, and GPX-1. However, in the presence of both Anisomycin and ethanol, the above effects of Gla were substantially diminished and even disappeared. Meanwhile, Gla failed to promote Nrf2 nuclear translocation and inhibit p-NF-κB nuclear translocation after Anisomycin treatment (Figure 8I–L). Taken together, we indicated that Gla may ameliorate the oxidative stress and inflammation caused by ethanol via the p38 MAPK/Nrf2/NF-κB pathway.

## 4. Discussion

Here, we explored the role of Gla on ethanol-fed mice, and cells and underlining mechanisms. Our results indicated that Gla improved ethanol-induced liver injury, lipid accumulation, inflammation, oxidative stress, and apoptosis. Mechanistically, we showed that Gla may regulate the NF-κB-mediated inflammation and the Nrf2-mediated oxidative stress by suppressing the p38 MAPK signaling pathway.

Drinking culture exists all over the world, and alcohol-induced liver injury is of great concern [27]. There is still no good drug for the treatment of ALD [28]. Gla is a kind of isoflavone that exists in the root of licorice [29], which possesses a variety of biological properties, such as anti-inflammatory [30], antioxidation [31,32], antineoplastic [33,34,35], anti-microbial [36], bone-protection [37], cardiovascular-protection, neuroprotection, liver protection, anti-obesity, and anti-diabetes [29], and has a certain influence in the field of food function and medicine. Gla is involved in many pathways, involving the NF-κB, MAPK, Wnt/b-catenin, and AMP-activated protein kinase pathways [29]. We proved that Gla can reduced liver injury in ALD mice, which not only further confirmed the hepatoprotective effect of Gla, but also provided a new direction for the clinical therapy of liver damage caused by excessive alcohol consumption.

In the early stage of alcoholic liver injury, NF-κB is activated, the key transduction molecule in the inflammation signal [38]. NF-κB nuclear translocation is a crucial step when NLRP3 transcription is initiated. In the quiescent state, cytosolic NF-κB exists in an inactive formation. IκBs kinase phosphorylates NF-κB, and the activated form of NF-κB translocates to the nucleus and combines its associated DNA motifs to induce the transcription of target genes [39]. Then, the NLRP3 inflammasome is activated, which activates caspase1 to allow IL-1β to mature and form the ASC complex [40], accompanied by the overexpression of inflammatory cytokines. In this study, Gla apparently decreased the NF-κB nuclear translocation and inactivated the NLRP3 inflammasome, thus decreasing the production of inflammatory cytokines. Gla has been reported to improve psoriasis-like inflammation in BALB/C mice by inhibiting the NF-κB pathway and other downstream inflammatory genes [41]. Consistent with our results, Gla showed significant anti-inflammatory effects. The activation of p38 MAPK promotes the phosphorylation of NF-κB [42], and we also proved that when the cells were treated with Anisamycin, a p38 MAPK agonist, Gla no longer significantly inhibited NF-κB activation, which meant that Gla may regulate inflammation by the p38 MAPK/NF-κB pathway.

Ethanol metabolism contributes to the accumulation of ROS through the CYP2E1 located in the endoplasmic reticulum, including hydrogen peroxide and superoxide anion O2^−^ [8]. These radicals rapidly combine with ethanol and then form hydroxyl radicals, ferrous oxides, or hydroxyethyl radicals, and so on, resulting in lipid peroxidation [6]. Gorce et al. proved that the overexpression of CYP2E1 was able to promote the aggravation of the alcoholic liver [43]. On the contrary, proteasome activity is increased, thus reducing alcoholic liver injury in CYP2E1^−/−^ mice [44]. However, our study showed that Gla could reduce the expression of CYP2E1 and the production of ROS in mice fed an ethanol diet. Therefore, CYP2E1 may be one of the targets of Gla treatment.

Oxidative stress, one of the foremost pathological factors of ALD, is highly associated with ROS. ROS causes oxidative stress directly, and antioxidant enzymes eliminate ROS effectively in the body [45]. However, chronic ethanol exposure leads to GSH depletion, decreases the antioxidant effect, and inhibits the activation of Nrf2, resulting in the over-accumulation of ROS [46], which is consistent with our results. Gla treatment significantly elevated the level of hepatic GSH and activated the hepatic SOD and CAT activities. It is worth mentioning that the Nrf2 pathway is a key defense mechanism for the response to oxidative stress [47]. We detected the effect of Gla on the Nrf2 pathway, and found that Gla could activate the nuclear translocation of Nrf2 to play an antioxidative role. A previous study showed that ROS production activated the p38 MAPK signaling pathway [48]. Our study not only found that Gla significantly inhibited the p38 MAPK, but also demonstrated that the activation of Nrf2 by Gla may be regulated through the p38 MAPK.

It is well-known that the MAPKs of mammals involve p38 MAPK, ERK, and JNK [49]. Thus, we also determined the expression of p-ERK and p-JNK, and illustrated that Gla did not affect the expression of p-ERK and p-JNK in the liver of ALD mice (Figure S1).

Although our study has achieved some results, there is still a key limitation. Our data only demonstrated the effect of Gla on the p38 MAPK at the cellular level, but the effect of the p38 inhibitor in the Gla-treated ALD animal model was lacking. In the future, we will design more precise and effective studies to better understand the mechanism of Gla.

## 5. Conclusions

In conclusion, drinking alcohol in the daily diet of human beings is common in the world, but it is not taken seriously, leading to an increasing number of ALD patients. We prove a protective effect of Gla against alcoholic liver injury for the first time, suggesting that Gla has the potential to be a dietary supplement or drug for ALD therapy. In addition, the protective effect of Gla is related to the p38 MAPK/Nrf2/NF-κB pathway.

## Figures and Tables

**Table 1:** The sequences of primers for qPCR analysis.

GENE	Forward Primer	Reverse Primer
h-GAPDH	GGTGGTCTCCTCTGACTTCAACA	GTTGCTGTAGCCAAATTCGTTGT
h-TNF-α	GTGACAAGCCTGTAGCCCAT	CAGACTCGGCAAAGTCGAGA
h-IL-1β	GACCTTCCAGGATGAGGACA	AGCTCATATGGGTCCGACAG
h-BCL2	TGTGTGTGGAGAGCGTCAAC	GAAATCAAACAGAGGCCGCAT
h-BAX	GAAGCTGAGCGAGTGTCTC	CAGACACGTAAGGAAAACGCA
h-SOD1	CCAGTGCAGGACCTCATTTT	TCATGGACCACCATTGTACG
h-SOD2	TCAATGGTGGGGGACATATT	GAACCTTGGACTCCCACAGA
m-Gapdh	GGAGAGTGTTTCCTCGTCCC	ACTGTGCCGTTGAATTTGCC
m-b-actin	CCCCTGAACCCTAAGGCCA	CGGACTCATCGTACTCCTGC
m-Nlrp3	CTACGGCCGTCTACGTCTTC	GGCCAAAGAGGAATCGGACA
m-Caspase1	ACTGCTATGGACAAGGCACG	GCAAGACGTGTACGAGTGGT
m-Asc	TGAGCAGCTGCAAACGACTA	CACGAACTGCCTGGTACTGT
m-Ho-1	GAAATCATCCCTTGCACGCC	TGTTTGAACTTGGTGGGGCT
m-Gclc	CATCTACCACGCAGTCAAGGA	GTTGGGGTTTGTCCTCTCCC
m-Gpx-1	AGTCCACCGTGTATGCCTTC	TTGCCATTCTGGTGTCCGAA
m-Bcl2	AGTACCTGAACCGGCATCTG	GGTATGCACCCAGAGTGATG
m-Bax	CACCTGAGCTGACCTTGGAG	CAGCCACCCTGGTCTTGG

## Data Availability

The data are available from the corresponding author upon reasonable requirement in the study.

## Supplementary Materials

The following supporting information can be downloaded: https://www.mdpi.com/article/10.3390/nu15092157/s1. Table S1: Composition and contents of Lieber–DeCarli diet components; Figure S1: p-ERK/ERK and p-JNK/JNK levels.
