# Supplementary material for: Glabridin Ameliorates Alcohol-Caused Liver Damage by Reducing Oxidative Stress and Inflammation via p38 MAPK/Nrf2/NF-κB Pathway
Source: Nutrients. 2023 Apr 30;15(9):2157. doi: 10.3390/nu15092157 (PMC10180694; doi:10.3390/nu15092157)
Supplement: Supplementary file 1 [file nutrients-15-02157-s001.zip › nutrients-2333102-supplementary.pdf]

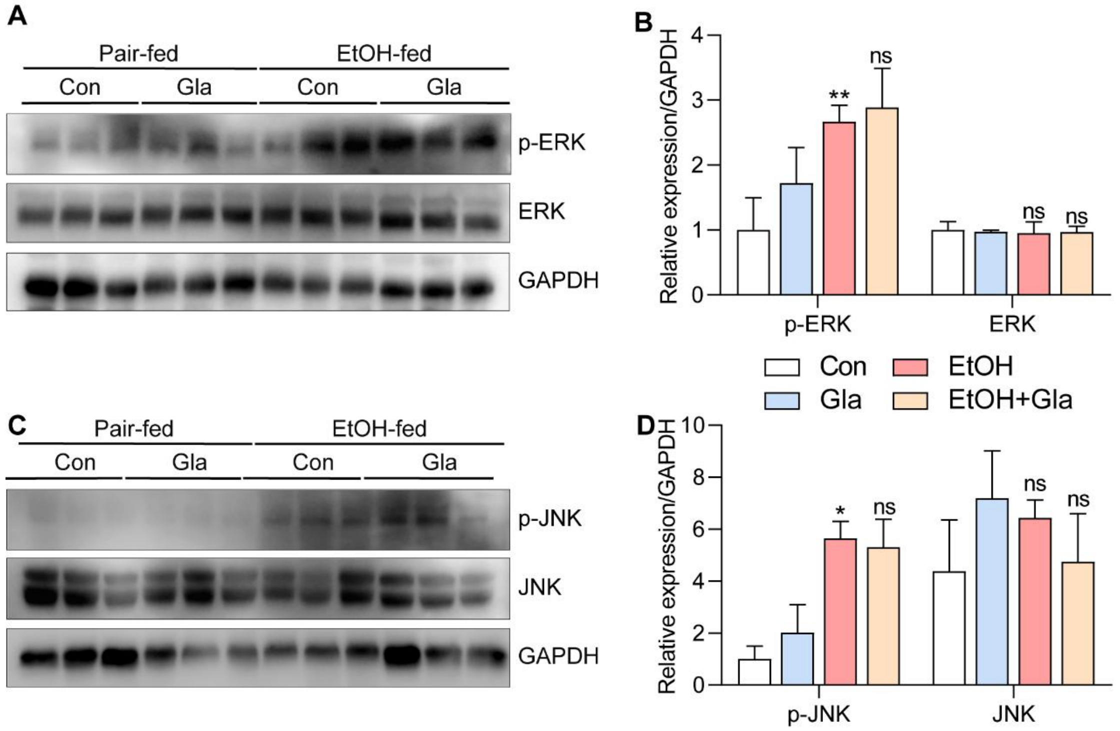

Figure S1. p-ERK/ERK and p-JNK/JNK levels in vitro. (**A**,**B**) Protein levels of p-ERK/ERK in the mouse liver were determined by Western blot (n = 3). (**C**,**D**) Protein levels of p-JNK/JNK in the mouse liver were determined by Western blot (n = 3). ns, not significant; * $p < 0.05$, ** $p < 0.01$ vs. "Con" group.

Table S1. Composition and contents of Lieber-DeCarli diet components (take for example the preparation of 1 L liquid diet).

Ingredient	Calories Per Unit (kcal/g)	Weight (g)	Calories (kcal)
Lieber-DeCarli ethanol mice diet			
Caisein	4	41.4	166
L-Cystine	4	0.5	2
DL-Methionine	4	0.3	1
Maltose Dextrin	4	25.6	102
Cellulose	0	10	0
Xanthan Gum	0	3	0
Corn Oil	9	8.5	77
Oliver Oil	9	28.4	256
Safflower Oil	9	2.7	24
Mineral Mix #210011	0.47	8.75	4
Vitamin Mix #310011	3.8	2.5	9
Choline Bitartrate	0	0.53	0
95% Ethanol	5.35	67 mL	358
Lieber-DeCarli regular control diet			
Caisein	4	41.4	166
L-Cystine	4	0.5	2
DL-Methionine	4	0.3	1
Maltose Dextrin	4	115.2	461

Cellulose	0	10	0
Xanthan Gum	0	3	0
Corn Oil	9	8.5	77
Oliver Oil	9	28.4	256
Safflower Oil	9	2.7	24
Mineral Mix #210011	0.47	8.75	4
Vitamin Mix # 310011	3.8	2.5	9
Choline Bitartrate	0	0.53	0
